# *QuickStats:* Age-Adjusted Percentage* of Adults Aged ≥65 Years,^†^ by Number of 10 Selected Diagnosed Chronic Conditions^§^ and Poverty Status — National Health Interview Survey, 2013–2015

**DOI:** 10.15585/mmwr.mm6607a6

**Published:** 2017-02-24

**Figure:**
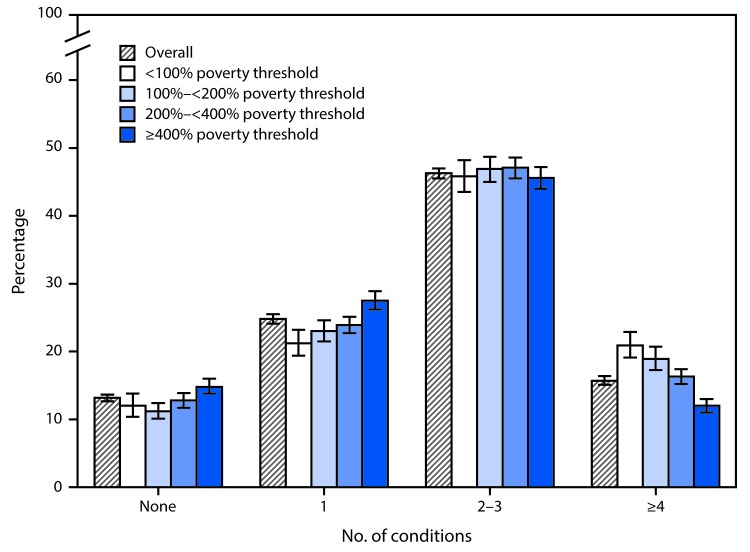
For the period 2013–2015, 13% of adults aged ≥65 years reported having none of 10 selected diagnosed chronic conditions; 25% had one, 46% had two or three, and 16% had four or more of the conditions. No differences by poverty status were observed among those who reported having two or three conditions, but those in the lowest income group (<100% of the poverty threshold) were less likely to have none or only one of the chronic conditions compared with those in the highest income group (≥400% of the poverty threshold). Those in the lowest income group also were more likely to have four or more conditions when compared with those in the highest income group (21% compared with 12%).

**Source:** National Health Interview Survey, 2013–2015. https://www.cdc.gov/nchs/nhis.htm.

